# Expression profile of tumour suppressor protein p53 and its regulator MDM2 in a cohort of breast cancer patients in a Tertiary Hospital in Ghana

**DOI:** 10.1371/journal.pone.0258543

**Published:** 2021-10-25

**Authors:** Francis Opoku, Kweku Bedu-Addo, Nicholas Akinwale Titiloye, Elijah Atta Manu, Charity Ameh-Mensah, Babatunde Moses Duduyemi

**Affiliations:** 1 Department of Physiology, Kwame Nkrumah University of Science and Technology, Kumasi, Ghana; 2 Department of Pathology, Kwame Nkrumah University of Science and Technology, Kumasi, Ghana; 3 Department of Pathology, University of Sierra Leone Teaching Hospitals Complex, Freetown, Sierra Leone; University of Limerick, IRELAND

## Abstract

**Background:**

Inactivation or mutation of the tumour suppressor gene *p53* or its regulator mouse double minute 2 (MDM2) is the commonest event in breast cancer. These altered genes usually express abnormally high levels of their proteins in many carcinomas. The phenotypic expression of p53 and MDM2 in breast cancer cases in our setting is not known. This study investigated the expression of the tumour suppressor protein p53 and its regulator MDM2, using immunohistochemistry in a Ghana breast cancer cohort.

**Method:**

A 9-year retrospective cross-sectional study on archived tissue blocks–formalin fixed paraffin embedded tissue (FFPE) was carried out. Demographic data were abstracted. Based on complete clinical data and availability of FFPE archived blocks 203 cases were selected for tissue micro array (TMA) construction. The TMA sections were subjected to immunohistochemistry (IHC) (ER, PR, HER2, p53, and MDM2). Expression of p53 and MDM2 were related to grade and molecular subtypes.

**Results:**

The age ranged from 17 to 92 years (mean = 49.34 ± 13.74). Most of the cases were high grade; grade II (34.9%) and grade III (55.7%). Fifty-four percent of the cases were triple negative. Invasive ductal carcinoma no special type was the commonest histotype (87.1%). Thirty-six percent (36%) of the cases expressed p53. Significant associations were found between p53 overexpression and histological grade (p = 0.034), triple negative (p = 0.0333) and luminal B (p<0.01) tumors. Most cases (93.1%) were negative for MDM2 expression. Significant association was found between MDM2 and HER2 over-expression as well as Ki-67. There was no significant positive correlation between MDM2 and p53 co-expression (p>0.05).

**Conclusion:**

The elevated level of p53 expression in the aggressive breast cancer phenotypes (high histological grade and triple negative) in our cohort suggest that P53 elevation may be a poor prognostic marker in our setting. High expression of MDM2 in our cohort with high Ki67; also in cases with Her2/neu overexpression known with predictable poor prognosis in the absence of target therapy suggest MDM2 may be associated with aggressive biological behaviour in our breast cancer cases. The non-significant association of p53 and MDM2 expression in the same cases as also documented by previous studies suggest independent genetic pathway in tumourigenesis.

## Introduction

Breast cancer is a multifactorial anomaly caused by interplay of environmental, hormonal and genetic alterations and modifications and remains the most common cancer among females in terms of frequency and mortality globally [1–3]. Plethora of studies have reported racial and ethnic diversities in the biology and outcome of breast cancer with higher mortality in comparison to low incidence seen among Africans where breast cancer are associated with aggressive biology, higher histological grade, lympho-vascular invasion, lack of hormone receptors and recurrence [4–8].

African breast cancer cases have a high percentage of triple negative breast cancer (TNBC) subtype and conventional chemotherapy remains the only major means of treatment with a number of the cases having unfavourable response [9–12]. There is continual increasing interest in further research into biomarkers either through the use of immunohistochemistry or RNA-based technologies to individualize treatment and improve prognosis [13, 14].

Tumour suppressor gene *p53*, mapped to chromosome 17q13 with aberrant p53 proteins expression have been observed in about half of all tumours and has proven to be an independent negative prognostic marker in breast cancer. It functions to maintain genomic integrity by initiating cascade of events that lead to cell cycle arrest (at G1/S or G2/M checkpoint), DNA repair and in some cases programmed cell death; upon cellular perturbations such as DNA damage and oncogene activation [15].

Mouse double minute 2 (*MDM2*) gene, an E3 ligase proto-oncogene located on chromosome 12 continuously tag p53 with ubiquitin with subsequent proteasome degradation negatively regulates p53 and maintain it in low concentration in an unstressed cell. Upon activation of p53 during cellular stresses, inhibitory effect of MDM2 on p53 ceases [16]. Mice with MDM2 knockout demonstrated embryonic lethality, while those with both MDM2 and p53 deletion showed normal embryonic development, thus, demonstrating the critical negative regulation of MDM2 on p53 [17]. Although MDM2 amplifications is common in many cancers, the role it plays in prognosis has been contradictory with some studies reporting favourable prognosis while others assert otherwise [18, 19]. Despite accumulated evidence of their prognostic value, *p53* mutation status is rarely obtained for routine breast cancer diagnosis [20].

The main aim of this study was to investigate, using immunohistochemistry, the expression profiles of p53 and MDM2 in a Ghana breast cancer cohort and correlate findings with clinicopathological features as well as the molecular subtypes.

## Materials and methods

### Ethical approval

Approval was obtained from the Committee on Human Research, Publications and Ethics, KNUST School of Medicine and Dentistry (CHRPE/AP/417/18) and the Research and Development Unit, Komfo Anokye Teaching Hospital (REG NO: RD/CR18/203) on “Molecular profiling of breast cancer in Kumasi.

#### Study design and tissue samples

A retrospective, cross–sectional and descriptive study was employed to investigate the expression profiles of p53 and MDM2 on Formalin Fixed Paraffin Embedded (FFPE) breast cancer tissues that were obtained from patients seen at the Department of Pathology from January, 2009 to December, 2017. The study outlines the generation of a breast tumor tissue microarray which includes 203 cases of the 1,631 cases that were diagnosed between 2009–2017. Patient’s data including age, sex, histological diagnosis, tumour grade and lymphovascular invasion were abstracted.

All consecutive malignant cases seen within the study period were included while cases with missing patient records, missing or damaged tissue blocks and inconclusive diagnosis were excluded.

#### H&E slides preparation and review

Haematoxylin and Eosin (H&E) stained slides were made from the FFPE tissue blocks. The H&E slides prepared were reviewed jointly by two pathologists following the guidelines of the Royal College of Pathologist and the National Quality Assessment Service. Representative tumour areas were marked and clinicopathological data on the cases were confirmed or amended, where necessary.

#### TMA construction

TMAs were constructed using a manual TMA machine (Micatu MicaArray Gen. 4) based on slight modifications following manufacturer’s guideline. Recipient tissue blocks were made using a standard mould supplied with the tissue miroarrayer. Guided by TMA map, two cylindrical cores (1mm each) were punched out from the marked areas on the donor blocks and transferred into pre-punched holes in the recipient block. To ensure firm and uniform insertion of the tissue cores in the recipient block, they were placed under incandescent lamp for one hour.

#### Immunohistochemical staining

Microtome was used to cut about 3μm-thick sections from each recipient TMA block and spread onto SuperFrosted Plus slides. The slides were deparaffinised in xylene and rehydrated using graded series of ethanol (100%, 90%, 70%) diluted with tris buffered saline (TBS). This was followed by washing the slides in distilled water. The slides were then incubated in citrate buffer in a pressure cooker for antigen retrieval. Peroxidase methanol and casein solutions were used to block background and non-specific staining respectively. Immunohistochemical dilution for estrogen receptor (ER), progesterone receptor (PR), human epidermal growth factor receptor 2 (HER2), Ki-67, p53 and MDM2 were carried out following the manufacturer’s instructions as detailed in Table 1 and the optimized tissue sections were incubated respectively in the diluted primary antibodies. The sections were then immersed in secondary antibody conjugated with Peroxidase and Anti Peroxidase and later developed in diaminobenzidine tetrahydrochloride (DAB). They were subsequently counterstained in haematoxylin, dehydrated in increasing grades of alcohol (70%, 80%, 90%, 95% and 100%) and mounted in DPX Mountant.

**Table 1:** Various antibodies used in the study.

*Antibody*	*Clone*	*Pretreat*	*Dilution*	*Control*	*Company*	*Address*
ER	1D5	ER1/20	1:50	Breast CA	BioCare Medical	Concord, CA
PR	PgR 636	ER1/10	1:400	Endo/Myome	DAKO	Carpinteria, CA
HER-2	CB 11	ER1/20	RTU	Breast CA	DAKO	Carpinteria, CA
Ki-67	MIB-1	ER1/20	1:80	Tonsil	DAKO	Carpinteria, CA
CYCLIN-D1	SP4	ER2/20	1:40	88-13792-7A	Thermo Scientific	Grandy Island, NY
p53	DO-7		1:40	S95-13083	Dako	Carpenteria, CA
MDM2	SMP14	TRILOGY	1:200	Liposarcoma	Santa Cruz	Santa Cruz, CA

#### Scoring of IHC

TMA sections were assessed for the presence of positive reaction, pattern of staining and intensity of reaction. The tumours were grouped into the major molecular subtypes based on slight modifications of methods described by Carey et al. [21], triple negative (ER-, PR- HER2-), luminal A (ER+ and/or PR+, HER2-), luminal B (ER+ and/or PR+, HER2+) and HER2 enriched subtype (HER2+, ER-, PR-). The scoring was done using the ASCO/CAP guidelines [22]. Ki-67 expression was categorized as low (<6%), moderate (6–10%) and high (>10%) [23].

#### Statistical analysis

Data analysis was carried out using Statistical Package for Social Sciences (SPSS) version 23. Correlations between parameters were assessed using chi-square test as Spearman’s correlation coefficient (r). A 95% confidence interval was used. A p-value ≤ 0.05 at the 95% confidence level was considered statistically significant.

## Results

A total number of 1,631 breast cancer cases were seen in the study period with 203 cases meeting the inclusion criteria.

The cases were seen between ages 17 to 92 with the mean age being 49.34 (SD ±13.74). The age group with the highest frequency was 40–49 years (31%), while the lowest frequency was seen in the 10–19 years (0.05%). Invasive ductal carcinoma,(No special type) was the commonest histological variant (83.0%). Other histological variants are described in Table 2. Most of the cases were high grade; grade II (34. 9%) and grade III (55.7%) while low grade (Grade I) was 9.4%.

**Table 2:** Age and histological characteristics of patients with breast cancer.

*Age categories(years)*	*Number of cases*	*Percent(%)*
10–19	1	0.05
20–29	5	2.5
30–39	44	22.0
40–49	63	31.5
50–59	44	22.0
60–69	24	12.0
70–79	12	6.0
80–89	5	2.5
90 and above	2	1.0
Missing cases	3	-
Total	203	100.0
*Histological type*		
Invasive carcinoma NST	166	83.0
Ductal carcinoma in situ	9	4.5
Metaplastic carcinoma	6	3.0
Invasive lobular carcinoma	5	2.5
Mucinous carcinoma	5	2.5
Invasive papillary carcinoma	2	1.0
Medullary carcinoma	2	1.0
Others	5	2.5
Missing cases	3	-
Total	203	100.0
*Histological diagnosis*		
Grade I	14	9.4
Grade II	52	34.9
Grade III	83	55.7
Missing	54	-
Total	203	100.0

*Percentages were calculated on the number valid cases.

### Immunohistochemical staining of the various markers

**Fig 1:**
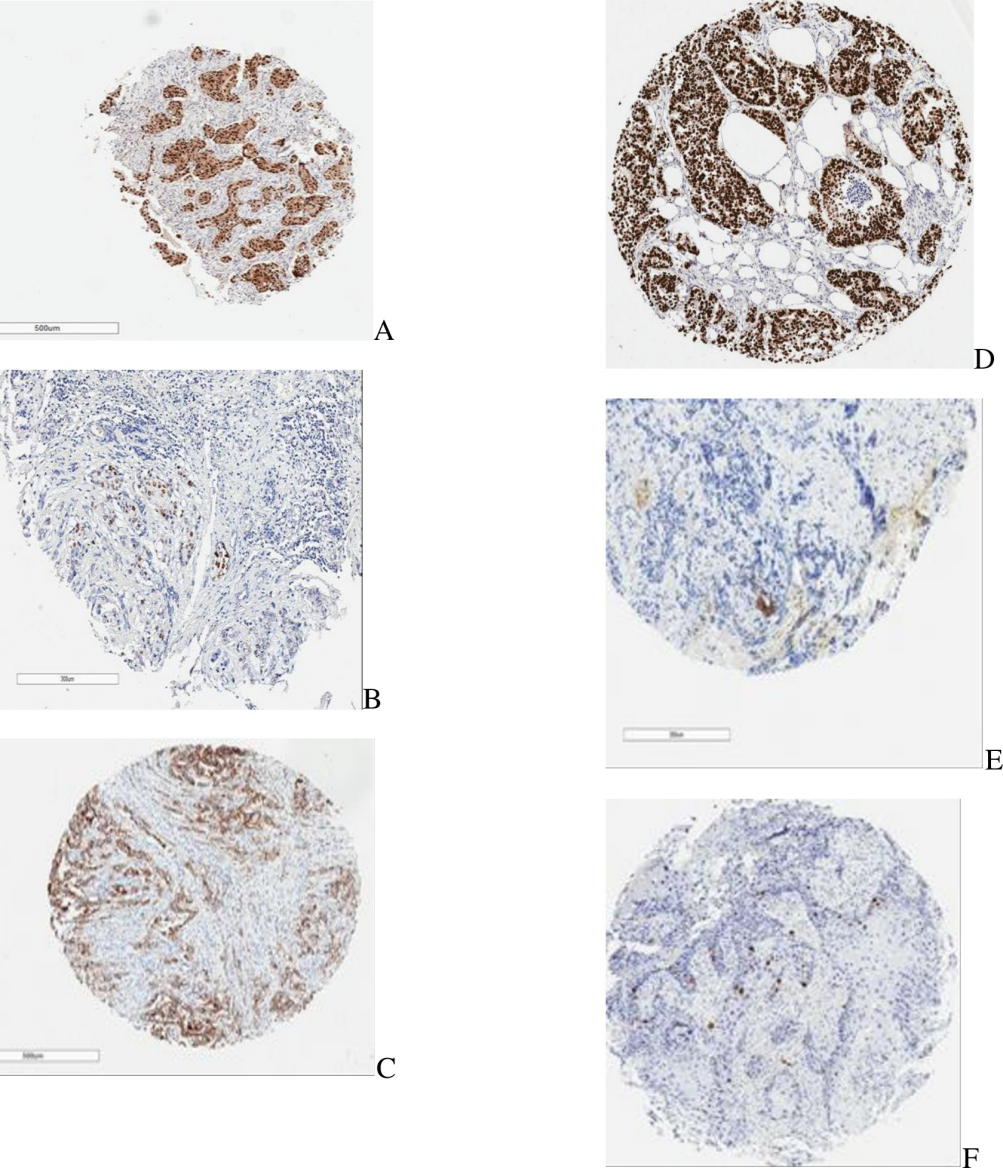
Sample photomicrographs of immunohistochemical stains for ER(A), PR(B), HER2(C), p53(D), MDM2(E) and Ki-67(F).

Majority of the cases were negative for ER, PR and HER2 expression (Table 3). 36.7 percent of the breast cancer cases were positive for p53 overexpression. MDM2 was overexpressed in 6.9% of the cases. Sixty two percent (62%) showed low expression of Ki-67 (Table 3). The molecular subtypes were also depicted in Table 3 with triple negative breast cancer cases accounting for more than half of the cases (54.4%). Fig 1 shows the photomicrographs of tissue cores of the various markers depicting positive stains from the various markers employed in the study.

**Table 3:** Immunohistochemical staining results on cases.

	*Number of cases*	*Percent (%)*
***ER***		
Positive	54	29.0
Negative	132	71.0
Missing	17	-
Total	203	100.0
***PR***		
Positive	20	10.9
Negative	163	89.1
Missing	20	-
Total	203	100.0
***Her2/neu***		
Positive	37	20.7
Negative	142	79.3
Missing	24	-
Total	203	100.0
***p53***		
Positive	65	36.7
Negative	112	63.3
Missing	26	-
Total	203	100.0
***MDM2***		
Positive	12	6.9
Negative	163	93.1
Missing	28	-
Total	203	100.0
***Ki-67***		
Low	113	62.1
Moderate	30	16.5
High	39	21.4
Missing	21	-
Total	203	100.0
***Molecular subtypes***		
Luminal A	32	19.7
Luminal B	16	9.9
HER 2 overexpression	26	16.0
Triple negative	88	54.4
Missing	41	-
Total	203	100.0

*Missing cases were lost to immunohistochemistry.

### Expression profiles of p53 and MDM2 with clinicopathological features

Associations between p53 and MDM2 with clinicopathological features were assessed using chi-squared text (Tables 4 and 5). Significant associations were found between p53 overexpression and histological grade (p = 0.034) and triple negative (p = 0.0333).

**Table 4:** Association of p53 with clinicopathological features, molecular subtypes and other biomarkers.

				p53 positives (%)	p53 negatives (%)	Chi-squared	p-value
	*Clinicopathological features*						
			<30	33.3	66.7		
Age categories			30–40	33.3	66.7		
			41–50	28.3	71.7	3.318	0.345
			>50	43.7	56.3		
Histological grade			Grade I	16.7	83.3	**6.781**	**0.034**
			Grade II	26.1	26.1		
			Grade III	45.7	54.3		
Lymphovascular invasion				42.9	57.1	2.094	0.245
	*Molecular subtypes*						
Luminal A				41.4	58.6	0.791	0.834
**Luminal B**				**86.7**	**13.3**	**15.709**	**<0.001**
Her2 overexpression				36.0	64.0	0.130	0.450
**Triple negative**				**31.0**	**69.0**	**4.651**	**0.033**
	*Other marker*						
**Ki-67**		Low		**27.7**	**72.3**	**11.957**	**<0.01**
		moderate		**41.4**	**58.6**		
		High		**59.5**	**40.5**		

*P-Value ≤ 0.05, considered as significant.

**Table 5:** Association of MDM2 with clinicopathological features, molecular subtypes and other biomarkers.

				MDM2 positives (%) (n = 12)		MDM2 negatives (%)		Chi-squared		p-value
	*Clinicopathological features*									
			<30	-		100.0		-		-
Age categories			30–40	4.3		95.7		1.885		0.597
			41–50	6.0		64.0				
			>50	10.0		90.0				
Histological grade			Grade I	7.7		92.3		1.276		0.528
			Grade II	2.3		97.7				
			Grade III	7.0		93.0				
Lymphovascular invasion				7.1		92.9		0.14		1.000
	*Molecular subtypes*									
Luminal A				3.6		96.4		0.751		0.691
Luminal B				12.5		87.5		0.755		0.323
**Her2 overexpression**				**12.0**		**88.0**		**1.036**		**0.027**
Triple negative				6.1		93.9		0.312		0.756
	*Other marker*									
Ki-67		Low		**2.0**	**98.0**		**9.084**		**0.011**	
		Moderate		**10.3**	**89.7**					
		High		**15.8**	**84.2**					

*P-Value ≤ 0.05, considered as significant.

Correlation between MDM2 and other parameters were also assessed. Significant positive correlation was found between MDM2 and HER2 overexpression and Ki-67 (Table 5).

#### Co-expression of p53 and MDM2

Chi-square test was used to assess co-expression of p53 and MDM2. MDM2 has 66.7% and 35.3% positive and negative cases respectively while p53 has 33.3% and 64.7% positive and negative cases respectively. There was no significant association between p53 and MDM2 co-expression (p = 0.06).

## Discussion

We analysed a total number of 203 cases with complete data set and retrievable blocks which accounted for 12.4% of the total breast cancer cases seen in the tertiary hospital over the study period. The clinicopathological demography shows age distribution of 17–92 years, mean age occurrence of 49.34 (SD ±13.74), highest age group of 40–49 years, predominance of Invasive ductal carcinoma NST (83%) and a high histological grade (Grade II 34.9% and Grade III 55.7%) which are all in agreement with previous studies in Ghana and environment [8].

The immunohistochemistry profile shows receptor positive breast cancer cases to be low (ER 29% and PR 10.9%). This is in agreement with Mensah *et al*. [8], and disagreement with Figueroa *et al*. [24] in earlier studies in Ghana. Titiloye *et al*. [7] on the disparity in the receptor positive breast cancer cases in African cases noticed a low pattern in studies conducted on archived breast cancer cases in comparison with prospective studies and our study followed the pattern of studies conducted on archival cases. This pattern is also reflected on our immunohistochemistry phenotypes with our study showing a predominance triple negative category. Our HER2 positive categories were similar to previous studies in our country [8].

Our study sought to understand the heterogeneous characteristics of breast cancer cases in a cohort with regards to histopathologic features, morphologic growth patterns and clinical behaviours [25] and immunohistochemical expression pattern of p53 and MDM2 [26]. This is novel as accumulated evidences of association of these oncogenic proteins with aggressive biologic tumour behaviours and treatment outcome [19, 27, 28], as seen in prior studies have not been replicated or dismissed in indigenous Ghana population.

In this study, p53 was over-expressed in 36.6% of the breast cancer cases in association with high histological grade in similarity with previous studies. Many of these studies also documented aggressive tumour behaviour, early disease recurrence and poor survival [15, 29]. However in our study we were unable to assess patient follow up details. Lost of patients with diagnosed malignancy to follow up during cancer therapy is a recurrent problem in our hospitals in West Africa due to a complex socio-economic, cultural and religious factors.

Also shown in previous studies were 12–84% of p53 expression in breast cancer cases depending on molecular subtype. The molecular subtype seen in association with p53 reported in this study is in agreement with previous studies of p53 positive breast cancer and molecular subtype [19, 26–29].

Generally, when cancerous cells express p53, it is indicative of the presence of mutant p53, hence, denoted as p53 positive [15, 19]. Unlike the wild type, mutant p53 protein is slowly degraded and therefore accumulate inside the cell making it a surrogate marker for mutated p53 [20].

Multiple studies investigating the prognostic significance of p53 also documented 20–30% somatic p53 mutation and an uncommon germline mutation [30]. A similar study involving over 1700 breast cancer patients describing the outcome of breast cancer reported twice higher mortality in tumours harbouring mutant p53 [31]. Demographic and genetic variations as well as sample sizes employed by the investigators were strongly associated with variation in p53 expressions in the studies.

In our study, p53 showed a significant association with high histological grade (p = 0.034). This finding is consistent with that observed by Dookeran *et al*. [26] in a study conducted among African-American women breast cancer cases.

Our findings showed a significant association between p53 and triple negative subtype (p = 0.033) in agreement with previous studies which have reported p53 expression correlating with triple negative with accompanying aggressive tumour behaviour, early age at diagnosis, high grade, metastasis, and poorer prognosis [26, 32]. This finding is **suggestive of** loss of p53 transcriptional functionality in maintaining genomic integrity by inducing apoptosis, cell cycle arrest, and senescence in order to halt progression of cancer in our breast cancer cases **with p53 overexpression [15].**

In this study, MDM2 overexpression was observed in 6.9% of the cases. This is in line with similar studies by Turbin *et al*. [33] who recorded 14% MDM2 expression out of which 10% showed weak MDM2 expression and 4% strong MDM2 expression. 6% MDM2 amplification in breast carcinoma has been reported in another study of over 2000 breast cancer cases [34]. These results were however, way below that observed by Loo *et al*. [19] who observed 80% MDM2 expression in a 787 multi-ethnic population based TMA study. MDM2 amplification and overexpression has been observed in many tumour types including breast cancer [16]. The role it plays in prognosis has been inconsistent, with MDM2 overexpression linked to both worst and favourable prognosis [18]. It is conceivable that this may be due to our limited comprehension of the role MDM2 plays in various ethnic and racial groups as well as multiple splice variants of the MDM2.

Our study found a significant association between MDM2 and HER2 overexpression subtype (p = 0.027) and is also in agreement with previous comprehensive analysis of the molecular subtype of breast cancer and MDM2 in which MDM2 expression were frequent in HER2 and luminal B subtypes but less common in luminal A and triple negative subtypes [12]. This study however showed no significant association between MDM2 and luminal B subtype.

Our study also found a significant positive correlation between MDM2 and a proliferative marker Ki-67, a mirrored result obtained by Turbin *et al*. [33].

In this study, association between p53 and MDM2 co-expression was not significant (p<0.05). Most studies reported that MDM2 overexpression and p53 mutation were not observed in the same cancer samples [35]. However Cordon-cardo *et al*. [36] and Turbin *et al*. [33] reported significant co-expression of p53 and MDM2. Thus, MDM2 and p53 alterations may not be mutually exclusive. Notwithstanding, they still concluded that MDM2 was an independent negative prognostic marker in breast cancer. In other words, the role of MDM2 in tumorigenesis is independent of its physiologic regulation of p53. Because p53 transcriptional function is deactivated by MDM2, co-expression of p53 and MDM2 might be unexpected in the same tumour samples. Thus our finding may be interpreted as increased activity of mutant p53-MDM2 or ‘gain of function’ of aberrant p53 proteins.

## Conclusion

The findings in this study are in line with earlier ones that showed overexpression of p53 to be associated with breast cancer with poor histological variant, high histological grade and poor immunohistochemical phenotype which are characteristics of breast cancer cases with aggressive biological behaviour.

High expression of MDM2 in our cohort with high Ki67; also in cases with Her2/neu overexpression known with predictable poor prognosis in the absence of target therapy suggest MDM2 may be associated with aggressive biological behaviour in our breast cancer cases.

The non-significant association of p53 and MDM2 expression in the same case as also documented by previous studies suggest independent genetic pathway in tumourigenesis.

A population based studies with expanded sample size and survival data will strenghten our hypothesis.

## References

[1] Sun YS, Zhao Z, Yang ZN, Xu F, Lu HJ, Shi W, et al. Risk factors and preventions of breast cancer. International Journal of Biological Sciences. 2017;13:1387–97. doi: 10.7150/ijbs.21635 29209143 PMC5715522

[2] Bray F, Ferlay J, Soerjomataram I, Siegel RL, Torre LA, Jemal A. Global cancer statistics 2018: GLOBOCAN estimates of incidence and mortality worldwide for 36 cancers in 185 countries. CA. Cancer J. Clin. 68:394–424. doi: 10.3322/caac.21492 30207593

[3] Traoré B, Koulibaly M, Diallo A, Bah M. Molecular profile of breast cancers in Guinean oncological settings. Pan Afr. Med. J. 2019; 33:1–7. doi: 10.11604/pamj.2019.33.22.18189 31312338 PMC6615767

[4] Kantelhardt EJ, Frie KG. How advanced is breast cancer in Africa? Lancet Glob. Heal. 2016;4:e875–6. doi: 10.1016/S2214-109X(16)30283-2 27855857

[5] Parada H, Sun X, Fleming JM, Williams-DeVane CR, Kirk EL, Olsson LT, et al. Race-associated biological differences among luminal A and basal-like breast cancers in the Carolina Breast Cancer Study. Breast Cancer Research. 2017;19:1–9. doi: 10.1186/s13058-016-0797-y 29228969 PMC5725885

[6] Duduyemi BM, Adegbola TA, Ipadeola O, Mortimer G, Salman R, Ulman V, et al. Expression of Oestrogen Receptors α and β in primary and recurrent breast cancers. J. Med. Med. Sci., 2014;5:25–36.

[7] Titloye NA, Foster A, Omoniyi-Esan GO, Komolafe AO, Daramola AO, Adeoye OA, et al. Histological Features and Tissue Microarray Taxonomy of Nigerian Breast Cancer Reveal Predominance of the High-Grade Triple-Negative Phenotype. Pathobiology. 2016; 83:24–32. doi: 10.1159/000441949 26730581

[8] Mensah AC, Yarney J, Nokoe SK, Opoku S, Clegg-Lamptey JN. Survival Outcomes of Breast Cancer in Ghana: An Analysis of Clinicopathological Features. OALib, 2016; 3:1–11.

[9] Opoku SY, Benwell M, Yarney J. Knowledge, attitudes, beliefs, behaviour and breast cancer screening practices in Ghana, West Africa. Pan Afr. Med. J. 2012;11:28. 22514762 PMC3325066

[10] Pace LE, Shulman LN. Breast Cancer in Sub-Saharan Africa: Challenges and Opportunities to Reduce Mortality. Oncologist. 2016;21(6):739–44. doi: 10.1634/theoncologist.2015-0429 27091419 PMC4912363

[11] Kumar P, Aggarwal R. An overview of triple-negative breast cancer. Arch. Gynecol. Obstet. 2016;293:247–69 doi: 10.1007/s00404-015-3859-y 26341644

[12] The Cancer Genome Atlas Network. Comprehensive molecular portraits of human breast tumours. Nature. 2012;490:1–10 doi: 10.1038/nature11412 23000897 PMC3465532

[13] Lacroix M, Toillon R, Leclercq G. p53 and breast cancer, an update. Endocrine-Related Cancer. 2006;13:293–326. doi: 10.1677/erc.1.01172 16728565

[14] Issaeva N. p53 Signaling in Cancers. Cancers (Basel). 2019;11:332. doi: 10.3390/cancers11030332 30857153 PMC6468470

[15] Miedl H, Lebhard J, Ehart L, Schreiber M. Association of the MDM2 SNP285 and SNP309 genetic variants with the risk, age at onset and prognosis of breast cancer in central european women: A hospital-based case-control study. Int. J. Mol. Sci. 2019;20:509 10.3390/ijms20030509 PMC6387136 30691044

[16] Oliner JD, Saiki AY, Caenepeel S. The role of MDM2 amplification and overexpression in tumorigenesis. Cold Spring Harb. Perspect. Med., 2016;6:1–16. doi: 10.1101/cshperspect.a026336 27194168 PMC4888815

[17] Jones SN, Roe AE, Donehower LA, Bradley A. Rescue of embryonic lethality in Mdm2-deficient mice by absence of p53. Nature. 1995;378:206–8. doi: 10.1038/378206a0 7477327

[18] Onel K, Cordon-cardo C. MDM2 and Prognosis. Molecular Cancer Research.2004;2:1–8 14757840

[19] Loo LWM, Gao C, Shvetsov YB, Okoro DR, Hernandez BY, Bargonetti J. MDM2, MDM2-C, and mutant p53 expression influence breast cancer survival in a multiethnic population. Breast Cancer Res. Treat. 2018;174:257–69. doi: 10.1007/s10549-018-5065-7 30470976 PMC6530987

[20] Hashmi AA, Naz S, Hashmi SK, Hussain ZF, Irfan M, Khan EY, et al. Prognostic significance of p16 & p53 immunohistochemical expression in triple negative breast cancer. BMC Clin. Pathol.2018;18:1–11. doi: 10.1186/s12907-017-0068-6 30305801 PMC6171321

[21] Carey LA, Perou CM, Livacy CA, Dressler LG, Cowan D, Conway K, et al. Race, breast cancer subtypes and survival in the Carolina Breast Cancer Study. JAMA. 2006;295:2492–502 doi: 10.1001/jama.295.21.2492 16757721

[22] Wolff AC, Hammond MEH, Allison KH, Harvey BE, Mangu PB, Bartlett JMS, et al. Human Epidermal Growth Factor Receptor 2 Testing in Breast Cancer. (American Society of Clinical Oncology/College of American Pathologists Clinical Practice Guideline Focused Update) Arch Pathol Lab Med. 2018;142:1364–1382; doi: 10.5858/arpa.2018-0902-SA 29846104

[23] Abubakar M, Orr N, Daley F, Coulson P, Raza Ali H, Blows F. Prognostic value of automated KI67 scoring in breast cancer: a centralised evaluation of 8088 patients from 10 study groups. Breast Cancer Res 18, 104 (2016). 10.1186/s13058-016-0765-6 27756439 PMC5070183

[24] Figueroa J, Lynn BC, Edusei L, Titiloye NA, Adjei E, Clegg-Lamptey JN, et al. Reproductive Factors and Risk of Breast Cancer by Tumour subtypes among Ghanaians women: A- population- based- case -control study,” International Journal of Cancer. 2020. 10.1002/ijc.32929 PMC8380990 32068253

[25] Rakha EA, Reis-Filho JS, Ellis IO. Combinatorial biomarker expression in breast cancer. Breast Cancer Res. Treat. 2010;120:293–308, doi: 10.1007/s10549-010-0746-x 20107892

[26] Wade M, Wang YV, Wahl GM. The p53 orchestra: Mdm2 and Mdmx set the tone. Trends in Cell Biology. 2010;20:299–309. doi: 10.1016/j.tcb.2010.01.009 20172729 PMC2910097

[27] Dookeran KA, Dignam JJ, Ferrer K, Sekosan M, McCaskill-Stevens W, Gehlert S. p53 as a Marker of Prognosis in African-American Women with Breast Cancer. Ann. Surg. Oncol. 2010;17:1398–405. doi: 10.1245/s10434-009-0889-3 20049641

[28] Ahmed HG, Al-adhraei MA, Al-thobhani AK. Correlations of Hormone Receptors (ER and PR), Her2/neu and p53 Expression in Breast Ductal Carcinoma Among Yemeni Women. The open Cancer Immunology Journal. 2011;4:1–9

[29] Al-Moundhri M, Nirmala V, Al-Mawaly K, Ganguly S, Burney I, Rizvi A, et al. Significance of p53, Bcl-2, and HER-2/neu Protein Expression in Omani Arab Females with Breast Cancer. Pathology and Oncology Research. 2003;9:226–31. doi: 10.1007/BF02893382 14688828

[30] Petitjean A, Achatz MIW, Borresen-dale AL, Hainaut P, Olivier M. TP53 mutations in human cancers: Functional selection and impact on cancer prognosis and outcomes. Oncogene. 2007;26:2157–65. doi: 10.1038/sj.onc.1210302 17401424

[31] Oliver M, Langer A, Carrieri P, Bergh J, Klaar S, Eyfjord J, et al. The clinical value of somatic TP53 gene mutations in 1,794 patients with breast cancer. Clinical Cancer Research. 2006;12:1157–67. doi: 10.1158/1078-0432.CCR-05-1029 16489069

[32] Wang XY, Liu Q, Sun JJ, Zuo WS, Hu Dw, Ma SG, et al. Correlation between p53 and epidermal growth factor receptor expression in breast cancer classification. Genet. Mol. Res 2015;14:4282–90. doi: 10.4238/2015.April.28.10 25966200

[33] Turbin DA, Cheang MC, Bajdik CD, Gelmon KA, Yorida E, De Luca A, et al. MDM2 protein expression is a negative prognostic marker in breast carcinoma. Mod. Pathol. 2006;19:69–74. doi: 10.1038/modpathol.3800484 16258514

[34] Al-Kuraya K, Schraml P, Torhorst J, Tapia C, Zaharieva B, Novotny H, et al. Prognostic relevance of gene amplifications and coamplifications in breast cancer. Cancer Res.2004;64:8534–40 doi: 10.1158/0008-5472.CAN-04-1945 15574759

[35] Manfredi J. The Mdm2– p53 relationship evolves: Mdm2 swings both ways as an oncogene and a tumor suppressor. Genes &Development. 2010;24:1580–9 doi: 10.1101/gad.1941710 20679392 PMC2912554

[36] Cordon-cardo C, Latres E, Drobnjak M, Olivia MR, Pollack D, Woodruff JM, et al. Molecular Abnormalities of mdm2 and p53 Genes in Adult Soft Tissue Sarcomas. Cancer research. 1994;54:794–9. 8306343

